# Ligand-Dependent Intramolecular Motion of Native Nicotinic Acetylcholine Receptors Determined in Living Myotube Cells via Diffracted X-ray Tracking

**DOI:** 10.3390/ijms241512069

**Published:** 2023-07-28

**Authors:** Koichiro Oishi, Mayu Nagamori, Yasuhiro Kashino, Hiroshi Sekiguchi, Yuji C. Sasaki, Atsuo Miyazawa, Yuri Nishino

**Affiliations:** 1 Graduate School of Sciences, University of Hyogo, 3-2-1 Kouto, Kamigori-cho, Ako-gun, Kobe 678-1297, Hyogo, Japan; rl18m001@stkt.u-hyogo.ac.jp (K.O.); kashino@sci.u-hyogo.ac.jp (Y.K.); 2 Center for Synchrotron Radiation Research, Japan Synchrotron Radiation Research Institute, 1-1-1 Kouto, Sayo-cho, Sayo-gun, Sayo 679-5198, Hyogo, Japan; sekiguchi@spring8.or.jp (H.S.); ycsasaki@edu.k.u-tokyo.ac.jp (Y.C.S.); 3 Graduate School of Frontier Sciences, The University of Tokyo, 5-1-5 Kashiwanoha, Kashiwa 277-8561, Chiba, Japan; 4 AIST-UTokyo Advanced Operando-Measurement Technology Open Innovation Laboratory, National Institute of Advanced Industrial Science and Technology, 6-2-3 Kashiwanoha, Kashiwa 277-0882, Chiba, Japan

**Keywords:** nicotinic acetylcholine receptor, diffracted X-ray tracking, intramolecular motion, ligand-dependent gating, living cell

## Abstract

Nicotinic acetylcholine receptors (nAChRs) are ligand-gated ion channels that play an important role in signal transduction at the neuromuscular junction (NMJ). Movement of the nAChR extracellular domain following agonist binding induces conformational changes in the extracellular domain, which in turn affects the transmembrane domain and opens the ion channel. It is known that the surrounding environment, such as the presence of specific lipids and proteins, affects nAChR function. Diffracted X-ray tracking (DXT) facilitates measurement of the intermolecular motions of receptors on the cell membranes of living cells, including all the components involved in receptor function. In this study, the intramolecular motion of the extracellular domain of native nAChR proteins in living myotube cells was analyzed using DXT for the first time. We revealed that the motion of the extracellular domain in the presence of an agonist (e.g., carbamylcholine, CCh) was restricted by an antagonist (i.e., alpha-bungarotoxin, BGT).

## 1. Introduction

The nAChR is a ligand-gated ion channel that is indispensable for signal transduction at the neuromuscular junction (NMJ), the synapse between a motor nerve terminal and a muscle cell. The nAChR is a member of the Cys-loop ligand-gated ion channel superfamily, which includes many essential receptors, including the 5-hydroxytryptamine type-3 receptor (5-HT_3_R), the gamma-aminobutyric acid type-A receptor (GABA_A_R), and glycine receptors (GlyRs) [1]. Acetylcholine (ACh) released from motor nerves binds to the extracellular domain of nAChRs present on the surface of a muscle cell and evokes conformational changes in the nAChRs. Movements of the extracellular domain caused by these conformational changes alter the channel domain and open the ion channel [2,3]. In this study, we term such local movements within a protein “intramolecular motion.” The ACh-dependent intramolecular motion of the nAChR leads to conformational changes between closed, open, and desensitized states. The study of intramolecular motion can improve our understanding of the mechanisms involved in channel open–closed transitions, i.e., in signal transduction across the NMJ.

The intramolecular motions involved in channel open–closed transitions have been studied through comparisons of the structures of each functional state determined using X-ray crystallography or cryo-electron microscopy (cryo-EM). In recent years, cryo-EM has become highly accurate, and various structures involving Cys-loop receptors reconstituted into lipid bilayers of nano-discs have been determined. These include serotonin-bound and apo 5-HT_3_Rs; benzodiazepine- and various anesthetic-bound GABA_A_Rs; glycine-, allosteric modulator-bound, and apo GlyRs; antagonist-, agonist-, and allosteric modulator-bound neural-type nAChRs; and the antagonist-bound muscle-type nAChR of *Torpedo californica* [4,5,6,7,8].

Reports have indicated that cholesterol and negatively charged lipids are required for nAChR activity [9,10,11,12,13,14,15], and recently elucidated atomic structures of Cys-loop receptors have revealed that cholesterol molecules occupy spaces between the transmembrane helices and interact with the receptor to modulate channel properties [4,5,16,17]. It was also reported that the cytoskeleton is related to channel activity [18]. Moreover, Unwin demonstrated that channel activation spreads from an nAChR via interactions among receptors that form a “functional unit” at the postsynaptic membrane [19].

Analyses of nAChRs embedded in the cell membrane with intact cytoskeletons are required to elucidate how lipids and the cytoskeleton are related to the genuine channel-gating mechanisms of nAChRs. It remains difficult to analyze the protein structure in situ because X-ray crystallography needs crystallization of the protein and cryo-electron microscopy needs purified proteins to average the large number of classified images in various directions. On the other hand, diffracted X-ray tracking (DXT) enables investigation of the conformational dynamics of functional proteins embedded in the cell membranes of living cells to elucidate channel-gating mechanisms [20,21]. DXT is a technique used to measure the intramolecular motion of a target protein by monitoring the motion of diffraction spots on gold nanocrystals labeled on the protein surface [22,23,24]. These intramolecular motions can be analyzed in two different axis views, i.e., tilting (θ direction) and twisting (Χ direction) motions. DXT does not need a number of purified protein molecules because it is a single-molecule analysis; therefore, it is possible to analyze the intramolecular motion in situ. 

In a previous work, we used the DXT method to analyze the ligand-dependent intramolecular motions of nAChRs embedded in the lipid bilayers of a proteo-liposome prepared from *Torpedo* electric organs [25]. The electric organ contains abundant nAChR-rich postsynaptic membranes and has been used extensively for structural and functional analyses of nAChRs [2,25,26]. nAChR-rich liposomes were prepared without any detergents. Therefore, the obtained nAChRs should still retain rapsyn, a key scaffold protein of nAChRs, as well as other proteins and boundary lipids. However, most of the cytoskeleton and the “functional unit” was dissociated following tissue homogenization. 

To analyze the genuine ligand-dependent intramolecular motion of nAChRs at intact postsynapses, we measure the motion of an nAChR using living C2C12-cell-derived myotube cells. C2C12-cell-derived mouse myotube cells are a well-established model for mammalian postsynapse of the NMJ [27,28,29]. On the cell membrane of these myotube cells, nAChRs and related proteins (i.e., rapsyn, MuSK, and LRP4) are densely packed and form clusters, which are also seen at the postsynapse of the NMJ in vivo [30,31,32]. It has been also confirmed that, in C2C12-cell-derived myotube cells, ACh provokes an intracellular Ca^2+^ increase that leads to muscle contraction, just as it does in vivo [33,34].

Specific labeling of a gold nanocrystal to the target protein and X-ray irradiation are key parts of the DXT method. In this study, gold nanocrystals are conjugated with Fab’ fragments of an anti-nAChR monoclonal antibody. By digesting Immunoglobulin G (IgG) to the Fab’ fragment, the orientation of an antibody against a gold nanocrystal can be controlled because the exposure of two cysteine residues at a site opposite to the antigen recognition site of Fab’ permits the binding of gold nanocrystals via a covalent gold–sulfur bond (instead of electrostatic interactions). Specific labeling of antibody-conjugated gold nanocrystals (“Ab-AuNCs”) to nAChRs present on the surface of myotube cells is confirmed with correlative light and electron microscopy. 

X-ray irradiation produces free radicals, which cause cell membrane erosion and cell death [35]. It is also known that low temperatures can reduce radiation damage to biological specimens [36,37,38]. Therefore, we use a temperature-controlled chamber to keep the myotube cells at 4 °C during X-ray irradiation. The X-ray-irradiated cells are then stained with propidium iodide, and we confirm that the cells remained alive during X-ray irradiation for DXT. Thus, for the first time, we are able to use DXT to measure the intramolecular motions of nAChRs expressed and clustered on the cell membranes of living myotube cells.

## 2. Results

### 2.1. Labeling of nAChRs with Ab-AuNCs

**Figure 1:**
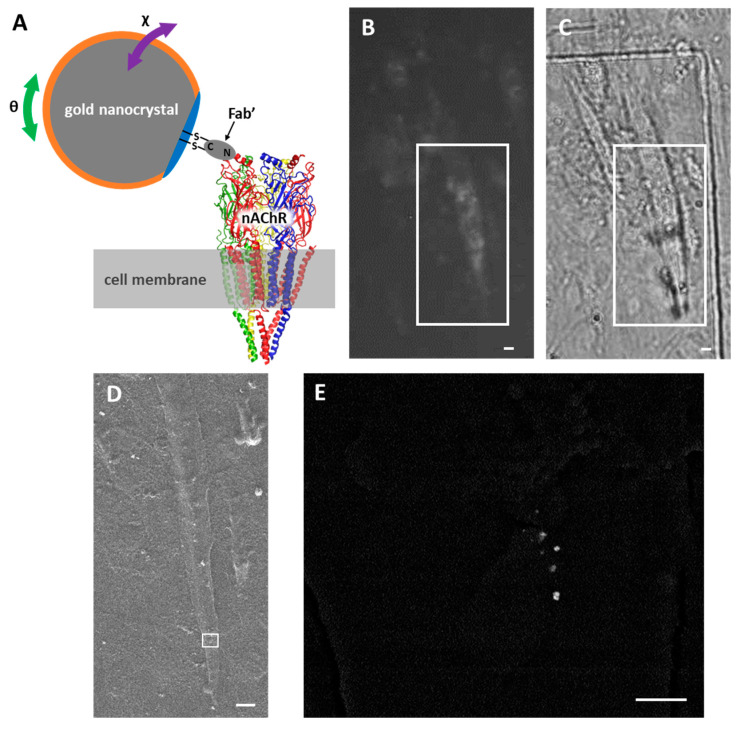
Labeling of nAChRs with Ab-AuNCs. (**A**) Schematic representation of an nAChR labeled with Ab-AuNCs. The orange perimeter indicates a layer coated with 6-mercapto-1-hexanol. The blue perimeter indicates a layer coated with bovine serum albumin and polyethylene glycol. The green arrow indicates motion in the θ direction (i.e., vertical to the cell membrane), and purple arrows indicate motion in the χ direction (i.e., parallel to the cell membrane). (**B**–**E**) Fluorescent and Ab-AuNC-labeled nAChRs on the myotube cells. (**B**) nAChR clusters imaged using fluorescent microscopy. A fluorescently labeled nAChR cluster can be observed in the boxed area. (**C**) Outlines of myotube cells visualized with phase-contrast microscopy. (**D**) The myotube cell in the boxed areas in (**B**,**C**) was identified in an SEM image. (**E**) The boxed area in (**D**) was then enlarged, and several AuNCs were observed. Scale bars: 10 µm (**B**–**D**) and 1 µm (**E**).

For an accurate analysis of molecular dynamics with DXT, it is necessary to conjugate a gold nanocrystal specifically, as well as with a covalent bond, to a specific site on a specific protein of interest. Thus, we conjugated a Fab’ fragment of IgG to the extracellular domain of an nAChR α subunit to a gold nanocrystal via the C-terminus thiol groups of the Fab’ fragment. At the same time, we covered the surfaces of the gold nanocrystals with 6-mercapto-1-hexanol, bovine serum albumin, and polyethylene glycol to prevent non-specific adsorption of nanocrystals on the cell surface (Figure 1A).

To confirm whether Ab-AuNCs specifically bound to nAChRs on the surfaces of myotube cells, we added Ab-AuNCs and a fluorescent nAChR ligand (i.e., Alexa488-conjugated BGT) to the myotube cell culture and then observed the results using fluorescent and scanning electron microscopy (SEM), correlatively. Some punctate nAChR clusters were observed on a myotube cell in a fluorescent image (Figure 1B,C). Moreover, myotube cells observed using fluorescent microscopy were identified with SEM (Figure 1D). Several gold nanocrystals were detected only at the position where the fluorescent puncta were observed (Figure 1E), confirming that Ab-AuNCs specifically bound to nAChRs on myotube cells.

### 2.2. Evaluation of X-ray Irradiation Damage to Myotube Cells 

To analyze the intramolecular motion of intact nAChRs in living cells, we needed to ensure no fatal damage to the cells was caused by taking DXT measurements. Therefore, the damage to myotube cells caused by X-ray irradiation was evaluated under the same conditions as the DXT measurements.

**Figure 2:**
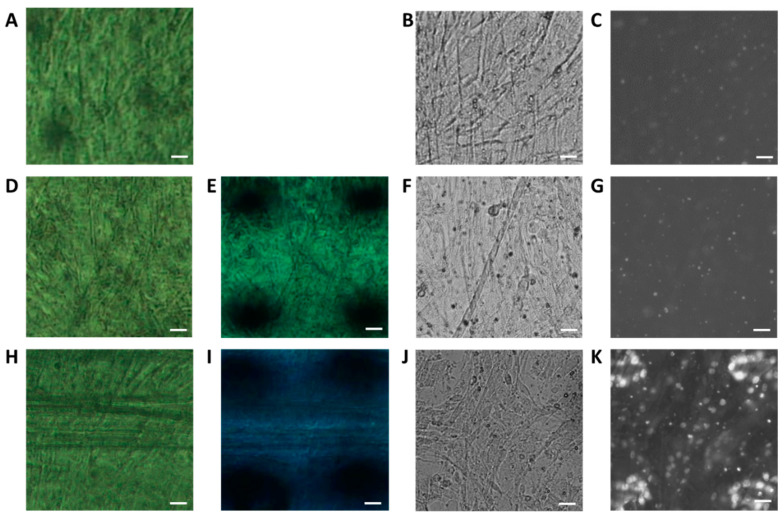
Evaluation of cell damage by X-ray irradiation. The myotubes on the polyimide film and a photosensitive film were set to the DXT holder, and phase-contrast images were observed both before (**A**,**D**,**H**) and after (**E**,**I**) X-ray irradiation. Next, the photosensitive film was removed and myotubes were stained with PI. Phase-contrast images (**B**,**F**,**J**) and PI-stained fluorescence images (**C**,**G**,**K**) were also observed. X-rays were irradiated under three conditions, including a negative control (no X-ray irradiation) (**A**–**C**), 10 ms of irradiation (**D**–**G**), and 100 ms of irradiation (**H**–**K**). Images shown in (**A**–**G**), and (**H**–**K**) depict the same respective areas. In the fluorescent images, the white oval dots of approximately 10 µm are the nuclei of dead cells (**C**,**G**,**K**). Scale bars: 20 µm.

Thus, myotube cells on a polyimide film were placed on a photosensitive film and irradiated with X-rays for 10 or 100 ms. A temperature-controlled chamber was used to keep the temperature at 4 °C to reduce X-ray damage to the cells. We evaluated the cells under three conditions: no X-ray irradiation (Figure 2A–C), 10 ms of irradiation (Figure 2D–G), and 100 ms of irradiation (Figure 2H–K). The myotube cells were observed using a phase-contrast microscope both before (Figure 2A,D,H) and after (Figure 2E,I) X-ray irradiation. Thereafter, the nuclei of dead cells were stained with PI and observed using a fluorescence microscope (Figure 2B,C,F,G,J,K).

After 10 ms of irradiation, we found no apparent changes in cell shape before and after X-ray irradiation (Figure 2A,B,D–F), nor did we find an increase in the number of dead cell nuclei compared to the negative control condition (Figure 2C,G). We noted that the white dots of a few micrometers in size present on all the fluorescent images (Figure 2C,G,K) were not the nuclei of dead cells since they were smaller than nuclei and had different shapes. After 100 ms of irradiation, however, we observed that cells on the edge of the irradiated area disappeared (Figure 2H–J), and many dead cell nuclei were observed in the irradiated area (Figure 2K). After 100 ms of irradiation, almost all the nuclei—not only in the irradiated area, but also in the vicinity—were stained with PI. Taken together, these results indicate that 100 ms of X-ray irradiation (113 kGy) induced cell death, but 10 ms of X-ray irradiation (11.3 kGy) did not cause significant damage to the cells. It was reported by a single-channel patch-clamp study [39] that the channel open time of muscle-type nAChRs was several milliseconds. Taken together, these results suggest that an X-ray irradiation time of 10 ms is sufficient to capture the open–closed motion of the channels.

### 2.3. DXT Analysis of nAChRs on Myotube Surface

**Figure 3:**
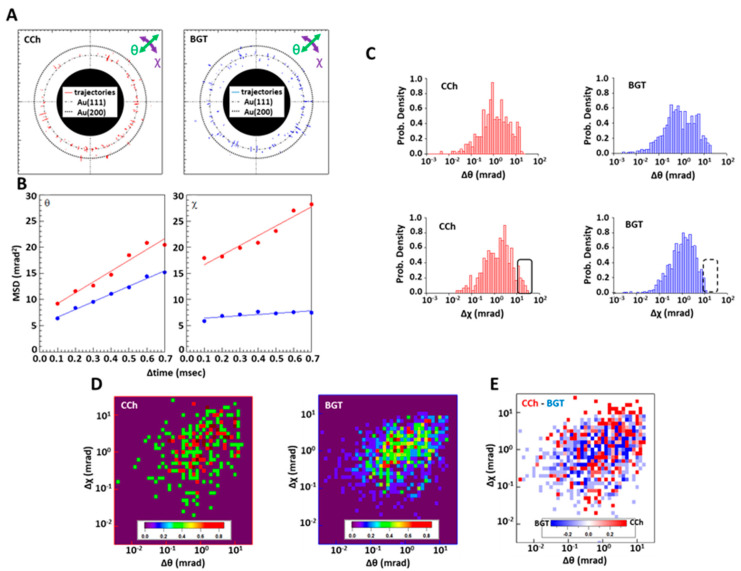
DXT analysis of nAChRs present on myotubes. (**A**) DXT traces of gold nanocrystals immobilized on the extracellular domain of an nAChR α subunit in the presence of CCh or BGT. The numbers of trajectories were 157 and 176 for CCh and BGT, respectively. The green arrows indicate movement in the θ (tilting) direction, and purple arrows indicate movement in the χ (twisting) direction. Chain-dashed lines show the expected position of Au (111), and dashed lines are the expected position of Au (200). (**B**) MSD curves in the θ and χ directions in the presence of CCh (red) and BGT (blue) obtained from the traces (**A**). (**C**) Distribution of the angular displacement. Motions more than 10 mrad in the χ direction in the presence of CCh were restricted by BGT (boxed area). (**D**) Intramolecular motion probability density maps of nAChRs in the presence of CCh and BGT. (**E**) Difference intermolecular motion maps of nAChRs in the presence of CCh and BGT. Characteristic motions in the presence of CCh and BGT are circled in red and blue, respectively.

The extracellular domain of an nAChR α subunit was labeled using Ab-AuNCs, and the single molecular motion over a period of 10 ms (i.e., 100 µs/frame) was analyzed with DXT following the addition of CCh (i.e., an agonist of nAChRs resistant to acetylcholine esterase) or BGT (i.e., an antagonist of nAChRs). Diffraction spots were observed, and most of them moved in different directions with different speeds (Supplementary Figure S1). The trajectories of the diffraction spots detected in the presence of CCh or BGT are shown in Figure 3A. These trajectories could be tracked for 0.7 ms on average. Most diffraction spots appeared surrounding the expected positions of Au (111) or Au (200). Their mean square displacement (MSD) curves in the θ and χ directions were then calculated using all the trajectories of Au (111) and Au (200), respectively (Figure 3A). In the θ direction, the slope and intercept of the MSD curve for the CCh addition were 20.70 ± 1.89 mrad^2^/ms and 7.143 ± 0.846 mrad^2^, respectively, while the slope and intercept for the BGT addition were 14.77 ± 0.55 mrad^2^/ms and 5.147 ± 0.246 mrad^2^, respectively. Moreover, in the χ direction, the slope and intercept of the MSD curve for the CCh addition were 18.45 ± 2.09 mrad^2^/ms and 14.796 ± 0.933 mrad^2^, respectively, while the slope and intercept of the MSD curve for the BGT addition were 2.311 ± 0.774 mrad^2^/ms and 6.206 ± 0.346 mrad^2^, respectively. These results show that movement in the χ direction was significantly suppressed by the addition of BGT compared to the addition of CCh. However, we found no significant difference in motion in the θ direction between samples added with CCh and BGT (Figure 3B). The distribution of each motion at Δt = 0.7 ms is shown in Figure 3C. In the χ direction, nAChR molecules with a large rotational motion of 10–100 mrad (i.e., the boxed area shown in Figure 3C) were seen following the addition of CCh but not BGT. In the θ direction, we found no significant differences between the additions of CCh or BGT (Figure 3C). Two-axis distribution maps at Δt = 0.7 ms and their subtraction maps are shown in Figure 3D,E. After subtracting BGT from CCh, a large motion population of 10–50 mrad (i.e., the circle shown in Figure 3E) was observed for CCh in the χ direction. This population also showed slightly greater motion in the θ direction, with about 1–10 mrad more than the population mean in the presence of BGT. Moreover, we also noted that trends seen in angular displacement (Figure 3C–E) were observed at different time intervals, ranging from 0.1 to 0.7 ms (Supplementary Videos S1 and S2).

## 3. Discussion

In this study, we captured the intramolecular motions of individual nAChR molecules on the cell membranes of living myotube cells—which are widely utilized for NMJ research as a postsynaptic model—in the presence of an agonist or antagonist using DXT.

In DXT, the movement of a target site of a protein is analyzed by measuring the rotation motion of a diffraction spot based on the position of a gold nanocrystal immobilized on the target site. When an X-ray with a wide bandwidth reaches the gold nanocrystal, a diffraction spot appears between the angle of the nanocrystal and the wavelength of the irradiated X-ray, which satisfies the Bragg condition. If the nanocrystal is then rotated by the intermolecular motion of the protein, where the nanocrystal is immobilized, the diffraction spot moves to the angular position between the current angle of the nanocrystal and the corresponding wavelength. 

The wider bandwidth of X-rays allows them to detect diffraction spots from gold nanocrystals with a greater variety of angles and to capture larger intramolecular motions of the target protein. Most intramolecular motions of proteins are complicated since they include movement in both the θ and χ directions. Therefore, X-rays with wider bandwidths are desirable for DXT. However, it was reported that X-rays with an energy width of 0.1 nm damaged living cells, while X-rays with an energy width of 0.02 nm were sufficiently low-energy so as to not affect cellular viability despite the fact that the photon flux of both was adjusted to 10^13^ photons/second [21]. In another previous study, ligand-dependent intramolecular motions of nAChRs in a proteoliposome from an electric ray were successfully measured using X-rays with an energy width of 0.08 nm (i.e., at a photon flux of 10^13^ photon/s) but not with an energy width of 0.02 nm [25].

It is also known that low temperatures reduce the radiosensitivity of biological tissues [38] and that there is a progressive decrease in radiosensitivity when enzymes are exposed to radiation at low temperatures [37]. In this study, the energy width was set to 0.08, the photon flux was 10^13^ photons/second, and the temperature of the experimental chamber was fixed at 4 °C. We then monitored cell damage following X-ray irradiation.

After 100 ms of X-ray irradiation, the myotube structures on the periphery of the X-ray-irradiated area disappeared. However, the structures seemed to be retained in the irradiated area (Figure 2J), although cell nuclei were strongly stained with PI, which is an indicator of cell death. In addition, lightly stained nuclei were distributed over a large area (Figure 2K). The nuclei of the cells were possibly stained outside of the X-ray irradiation area because myotube cells are multinucleated by cell fusion, and the lightly stained nuclei may show parts of these irradiated myotube cells. An amount of 10 ms of X-ray irradiation did not deform cell structures, and the number of PI-stained nuclei did not increase relative to non-irradiated myotube cells. Moreover, myotube cells exposed to 10 ms of X-ray irradiation at room temperature showed obvious deformation, and the experimental chamber being held at 4 °C may also have reduced X-ray-irradiation-induced cell damage. Patch-clamp studies revealed that the nAChR channel-opening time on myotube cells derived from C2C12 cells was less than 0.7 ms when CCh was added to the culture medium. Thus, even 10 ms is sufficient to capture the ligand-dependent channel opening and closing movement of nAChRs [40].

The MSD curves of the DXT measurements of nAChRs showed that motion in the χ direction was significantly suppressed by the addition of BGT (Figure 3B). These results are consistent with previous DXT measurements of nAChRs using proteoliposomes prepared from *Torpedo californica* electric organs [25]. A number of structural studies of nAChRs have demonstrated that the rotation of an α subunit in the χ direction serves as the driving force of ligand-dependent channel opening and closing [2,3,41,42,43]. In this study, rotation with an angle of more than 10 mrad in the χ direction was completely restrained by the presence of BGT (Figure 3C). The rotation blocked by BGT corresponded to 0.6°–1.7°. Furthermore, comparison between closed structures [Protein Data Bank ID: 2BG9] and open structures [Protein Data Bank ID: 4AQ9] showed that the rotation angle for the open–closed transition in the χ direction was 1.2° at the main immunologic region where the gold nanocrystal was conjugated via the Fab’ fragment. Therefore, movement with an angle of more than 10 mrad in the χ direction, as detected with DXT, may correspond to the rotational motion related to channel gating. This finding is consistent with the fact that this movement was restrained by BGT, which is a strong antagonist of nAChRs. We also observed small rotational motions with angles less than 10 mrad/ms, ranging from 0.0006° to 0.6°. These small motions were not restricted by BGT. A recent paper reported that some Cys-loop receptors fluctuate in the absence of ligands, meaning that there are several structures that correspond to each functional state [4,44,45,46,47,48]. Since the ion channel does not open in the presence of BGT, rotational motion with an angle of less than 10 mrad may not be related to channel function or may act as a functional warm-up for channel opening. Two-dimensional probability density maps of angular velocity indicate that molecular groups showing large movements in the presence of CCh that were also restrained in the presence of BGT ranged from 10 mrad to 50 mrad in the χ direction and from 1 mrad to 50 mrad in the θ direction (Figure 3D,E). These movements (Figure 3E) were slightly larger than the average in the presence of CCh (Figure 3E). This indicates that the direction of the rotation in the main immunologic region related to channel opening was approximately but not exactly parallel (χ direction) to the lipid bilayer. These movements were also detected when proteoliposomes from the *Torpedo californica* electric organ were used to measure the ligand-dependent motion of nAChRs with DXT. 

In this study, we successfully used DXT to capture the intramolecular motion of native nAChRs, ion channels that are associated with boundary lipids and proteins on the cell membranes of living postsynaptic model cells. Electrophysiological studies have shown that nAChR activity is regulated by their surrounding environment—e.g., the presence of cholesterol, acidic lipids, scaffold proteins, and mechanical stimuli against the plasma membrane [49,50,51].

In recent years, a number of membrane proteins have been solubilized from the lipid bilayer, purified, and reconstituted into nano-discs to solve their structures. However, producing intact complexes using nano-discs is not always possible since some interactions are very fragile and others involve interactions with as-yet-unknown proteins in vivo. The DXT method using intrinsic proteins in living cells is a promising technique for investigating the original motion and function of nAChRs. Moreover, a recent study suggested that Cys-loop receptors in resting state do not remain in a fixed and specific conformation but, rather, fluctuate [1,42,43,44,45,46]. In the future, DXT may be one of the most effective tools for analyzing the functional conformational changes in Cys-loop receptors, including fluctuations, which in turn can quantify the individual movements of individual protein molecules.

## 4. Materials and Methods

### 4.1. Cell Culture

C2C12 myoblast cells (ATCC, Manassas, VA, USA) were cultured on a gelatin-coated 7.5 µm thick polyimide film (Du Pont-Toray, Tokyo, Japan) or in a gridded µ-dish (Ibidi, Fitchburg, WI, USA) in Dulbecco’s Modified Eagle Medium (DMEM) (Sigma-Aldrich, St. Louis, MO, USA) including 10% fetal bovine serum (Gibco, Palo Alto, CA, USA) and a penicillin-streptomycin solution (Gibco) at 37 °C and 5% CO_2_. When the cells reached 100% confluency, the medium was changed to DMEM containing 5% horse serum (Gibco). After two or three days, myoblasts differentiated to myotubes, agrin (kindly donated by Dr. Shigemoto of the Tokyo Metropolitan Institute of Gerontology) was added to the medium, and cultivation was continued for 12–16 h.

### 4.2. Preparation of Ab-AuNCs 

Gold nanocrystals were obtained via epitaxial growth on a KCl (100) crystal substrate surface under a 10^−4^ Pa vacuum [22]. Anti-AChR antibody F(ab’)_2_ fragments were prepared via ficin digestion of anti-AChR antibody mAb35, which was purified from the supernatant of hybridoma cells (Developmental Studies Hybridoma Bank, Iowa city, IA, USA). Fab’-labeled gold nanocrystals with hydroxyl-terminated surfaces (Ab-AuNCs) were then prepared using the following procedure. After dehydration of gold-nanocrystal-grown KCl substrates by heating at 160 °C for five minutes, 10 mM of 6-mercapto-1-hexanol (451088, Sigma-Aldrich) dissolved in ethanol was applied to the surfaces of gold nanocrystal substrates and incubated in an airtight container filled with nitrogen gas overnight. Gold nanocrystals were then released from the KCl substrates with 500 µL of suspension buffer (i.e., 20 mM Tris-HCl, 10% glycerol, and 1% sodium citrate adjusted to pH 9) containing 21 µg of F(ab’)_2_ fragment. This gold nanocrystal suspension was then dialyzed in the same suspension buffer using Spectra/Por 7 (MW: 50 kDa) (Spectrum Labs, Rancho Dominguez, CA, USA) for 1 h at 4 °C. An additional 21 µg of F(ab’)_2_ fragment was added to the suspension, and it was sonicated on ice for one hour. Bovine serum albumin and polyethylene glycol (MW: 5000 kDa) were added to final concentrations of 0.5% and 1%, respectively, and the resulting mixture was sonicated on ice for 1 h.

### 4.3. Gold Nanocrystal Labeling of nAChRs

Ab-AuNCs were added to the myotube culture, and the mixture was incubated for one hour at 4 °C. For fluorescent microscopy, Alexa 488-conjugated BGT (Molecular probes, Eugene, OR, USA) was also added. Myotubes were then washed with DMEM and kept at 4 °C until subsequent DXT measurement.

### 4.4. Correlative Light and Electron Microscopy

nAChRs labeled with Alexa 488 and gold nanocrystals were observed with a fluorescent microscope (Axioplan 2, Carl Zeiss, Oberkochen, Germany) and then were fixed with 2% paraformaldehyde solubilized in PBS. The positions of the nAChR clusters were recorded using grid maps captured with phase contrast. After observation through fluorescent microscopy, clusters were further fixed with 2.5% glutaraldehyde for one hour and then dehydrated in graded ethanol and t-butanol. Samples were then freeze-dried with JFD-320 (JEOL, Tokyo, Japan). Gold nanocrystals at the fluorescent site were imaged using a JSM-6701F scanning electron microscope (JEOL).

### 4.5. Assessments of Cell Viability

Myotubes on the polyimide film were placed onto a cassette for DXT measurement with a photosensitive film (GAFCHROMIC Film HD-810, Ashland, Covington, KY, USA) enclosed in a plastic film. Myotubes were then observed with a phase-contrast microscope (CX41, Olympus, Tokyo, Japan). Next, the cassette was fixed in a temperature-controlled chamber adjusted to 4 °C. X-rays with an energy bandwidth of 0.08 (i.e., 15.8 keV peak energy at a photon flux of 10^13^ photons/second and a beam size of 0.05 mm in diameter) were irradiated for 10 or 100 ms at 36 individual positions (i.e., 6 × 6) within 1 mm^2^ (i.e., 1 mm × 1 mm) at BL40XU in SPring-8 (Hyogo, Japan). After X-ray irradiation, myotubes were again observed using a phase-contrast microscope. The polyimide film was removed from the cassette, and myotubes were stained with 0.6 µM propidium iodide for five minutes before being washed three times with Fluoro Brite DMEM (Gibco). The polyimide film was then placed on a glass slide and observed with Axioplan 2.

### 4.6. DXT

DXT measurements were conducted at BL40XU in SPring-8 (Hyogo, Japan). Time-resolved diffraction images of gold nanocrystals were recorded using an X-ray image intensifier (V7739P, Hamamatsu photonics, Hamamatsu city, Japan) and a CMOS camera (Phantom V2511, Vision Research, Wayne, NJ, USA) with incident X-rays, as described above. The distance between the sample and the detector was set to 50 mm. Amounts of 2 µM of carbamylcholine (nAChR channel open–close was detected on myotube cells under this concentration [52]) or 0.2 µM of BGT (sufficient concentration to keep nAChR channels closed [53]) were added to the myotube culture on the polyimide film just before or 30 min before measurement, respectively. The polyimide film was then placed onto a cassette for DXT measurement in a temperature-controlled chamber at 4 °C. For each polyimide film, diffractions at 36 positions were recorded. Recording occurred at 10 ms per position at a time resolution of 100 µs. Diffraction spots were tracked using TrackPy, version 0.4.1 (URL: https://doi.org/10.5281/zenodo.1226458 (accessed on 20 July 2023), and trajectories were analyzed using a custom script written within IGOR Pro (WaveMetrics, Portland, OR, USA).

## Data Availability

Not applicable.

## Supplementary Materials

The following supporting information can be downloaded at: https://www.mdpi.com/article/10.3390/ijms241512069/s1.
