# Supplementary material for: Ligand-Dependent Intramolecular Motion of Native Nicotinic Acetylcholine Receptors Determined in Living Myotube Cells via Diffracted X-ray Tracking
Source: Int J Mol Sci. 2023 Jul 28;24(15):12069. doi: 10.3390/ijms241512069 (PMC10418694; doi:10.3390/ijms241512069)
Supplement: Supplementary file 1 [file ijms-24-12069-s001.zip › Suppli/suppli.pdf]

Supplementary information

Ligand-dependent intramolecular motion of native nicotinic acetylcholine receptors in living myotube cells by diffracted X-ray tracking

Koichiro Oishi [1], Mayu Nagamori [1], Yasuhiro Kashino [1], Hiroshi Sekiguchi [2], Yuji C. Sasaki [2,3,4], Atsuo Miyazawa [1] and Yuri Nishino [1]

1. Department of Science, Graduate School of Sciences, University of Hyogo, 3-2-1 Kouto, Kamigori-cho, Ako-gun, Hyogo 678-1297, Japan; rl18m001@stkt.u-hyogo.ac.jp (K.O.); kashino@sci.u-hyogo.ac.jp (Y.K.); atsuo@sci.u-hyogo.ac.jp (A.M.); ynishino@sci.u-hyogo.ac.jp (Y.N.)
2. Center for Synchrotron Radiation Research, Japan Synchrotron Radiation Research Institute, 1-1-1 Kouto, Sayo-cho, Sayo-gun, Hyogo 679-5198, Japan; sekiguchi@spring8.or.jp
3. Graduate School of Frontier Sciences, The University of Tokyo, 5-1-5 Kashiwanoha, Kashiwa, Chiba 277-8561, Japan; ycsasaki@edu.k.u-tokyo.ac.jp
4. AIST-UTokyo Advanced Operando-Measurement Technology Open Innovation Laboratory, National Institute of Advanced Industrial Science and Technology, 6-2-3 Kashiwanoha, Kashiwa, Chiba 277-0882, Japan.

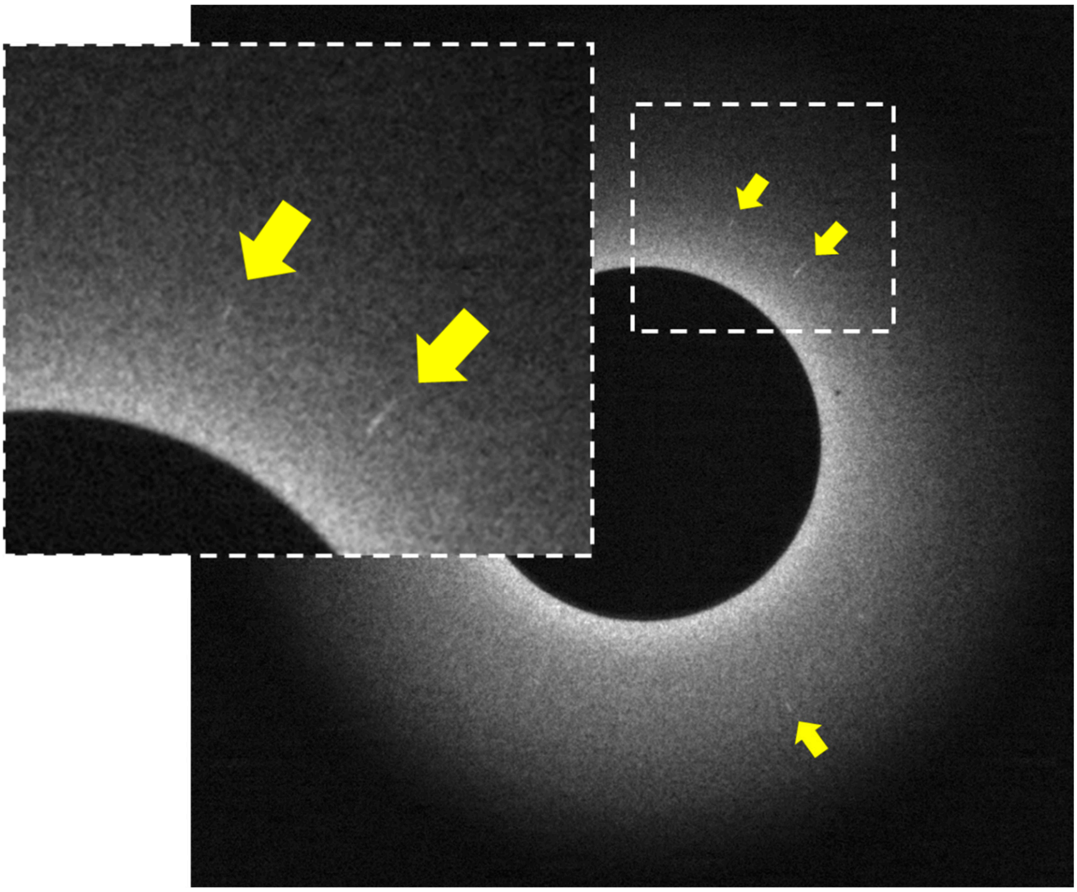

Supplementary Figure S1. Actual DXT diffraction image on nAChR with Ab-AuNCs. Yellow arrows indicate diffraction spots. The insert image at the upper left is an enlargement of the dashed square located at the upper right.

Supplementary Video S1. Distribution of the angular displacement of θ direction and χ direction which presence of CCh or BGT at various time intervals from Δt = 0.1–0.7 ms.

Supplementary Video S2. Two-axis distribution of the angular displacement which presence of CCh or BGT at various time intervals from Δt = 0.1–0.7 ms.
